# Incidental Intense Fibroblast Activation Protein Inhibitor (FAPI) Uptake in Bilateral Gluteal Myositis Ossificans: A Case Report

**DOI:** 10.7759/cureus.59520

**Published:** 2024-05-02

**Authors:** Rakan Al-Rashdan, Saad Ruzzeh, Nabeela Al-Hajaj, Ula Al-Rasheed, Akram Al-Ibraheem

**Affiliations:** 1 Nuclear Medicine, King Hussein Cancer Center (KHCC), Amman, JOR

**Keywords:** non-neoplastic, incidental, myositis ossificans, fapi, pet/ct

## Abstract

Positron emission tomography/computed tomography (PET/CT) using 18F-fluorodeoxyglucose ([18F]-FDG) is a widely adopted imaging modality for detecting hypermetabolic lesions. However, emerging positron-emitting tracers, such as radiopharmaceuticals featuring fibroblast activation protein (FAP) inhibitors (FAPI) labeled with [18F] or [68Ga], have opened new avenues in nuclear medicine. This case report focuses on the unique behavior of [68Ga]-FAPI in bilateral gluteal myositis ossificans, an infrequent condition characterized by soft tissue ossification. A 45-year-old woman with gastric adenocarcinoma underwent subtotal gastrectomy and received neoadjuvant and adjuvant chemotherapy; [68Ga]-FAPI PET revealed metastatic processes and unexpected [68Ga]-FAPI avid intramuscular ossifications in the pelvic and bilateral thigh muscles. Even though there was no history of trauma, the patient was diagnosed with myositis ossificans, a condition marked by non-cancerous ectopic ossifications. Diagnosis relies on history, radiology, and/or histology. FAPI imaging, increasingly used for inflammatory and infectious diseases, can exhibit uptake in benign conditions, including those involving bones and joints. This case report is the first to document incidental bilateral [68Ga]-FAPI uptake in bilateral gluteal myositis ossificans. The robust [68Ga]-FAPI activity in myositis ossificans highlights the importance of considering myositis ossificans in the context of soft tissue calcifications with intense [68Ga]-FAPI uptake.

## Introduction

Positron emission tomography/computed tomography (PET/CT) has emerged as a pivotal hybrid imaging modality, witnessing a remarkable surge in both utilization and application in recent years. The diagnostic technique primarily employs 18F-fluorodeoxyglucose ([18F]-FDG) as the radiopharmaceutical, which effectively detects hypermetabolic lesions due to their increased glycolytic metabolic activity [1].

In pursuit of metabolic pathways beyond glucose metabolism, various positron-emitting tracers have been proposed as viable alternatives to [18F]-FDG [2]. Among these, there is a burgeoning interest in radiopharmaceuticals containing fibroblast activation protein (FAP) inhibitors, referred to as FAPI, labeled with either [18F] or [68Ga]. These agents hold substantial promise as emerging tools in nuclear medicine [3,4].

FAPI compounds demonstrate an avidity for binding to FAP, a transmembrane serine protease exhibiting heightened expression in activated fibroblasts [5]. These activated fibroblasts are implicated in a wide array of pathophysiological contexts, including wound healing, inflammatory responses, and oncogenic processes, highlighting the versatility of FAPI as both a diagnostic and therapeutic avenue [6]. While FAPI utilization has surged in oncology to address tumors with limited FDG uptake, its role is also evolving in assessing inflammatory and infectious conditions, albeit with limited substantiated evidence in the existing literature [7-12].

Myositis ossificans (MO) is a rare pathological entity characterized by the formation of non-neoplastic heterotopic ossifications within soft tissues outside the skeletal framework. It may occur idiopathically or manifest within 4-12 weeks following a traumatic event [13].

In the context of this case report, we aim to underscore the significant observation of marked [68Ga]-FAPI uptake in the bilateral gluteal regions, ultimately diagnosed as MO. To our knowledge, there exists a dearth of case reports specifically addressing the distinct radiopharmaceutical behavior of [68Ga]-FAPI in bilateral gluteal MO.

## Case presentation

A 45-year-old female with an unremarkable medical and surgical history, and no familial cancer history, presented with recurrent vomiting episodes and was initially managed conservatively at a local hospital. Subsequently, she deteriorated, experiencing loss of consciousness due to severe dehydration and multiple electrolyte imbalances. Following treatment, the patient regained consciousness.

Gastroscopy showed a gastric tumor that was ulcerating and blocking the gastric outlet. A gastroduodenal stent had to be put in and a biopsy had to be done, which went smoothly. Histopathology identified a gastric poorly differentiated adenocarcinoma with signet ring features. CT scan showed pelvic cystic lesions and a right lung mid-zone nodule, prompting a referral to our tertiary cancer center.

**Figure 1:**
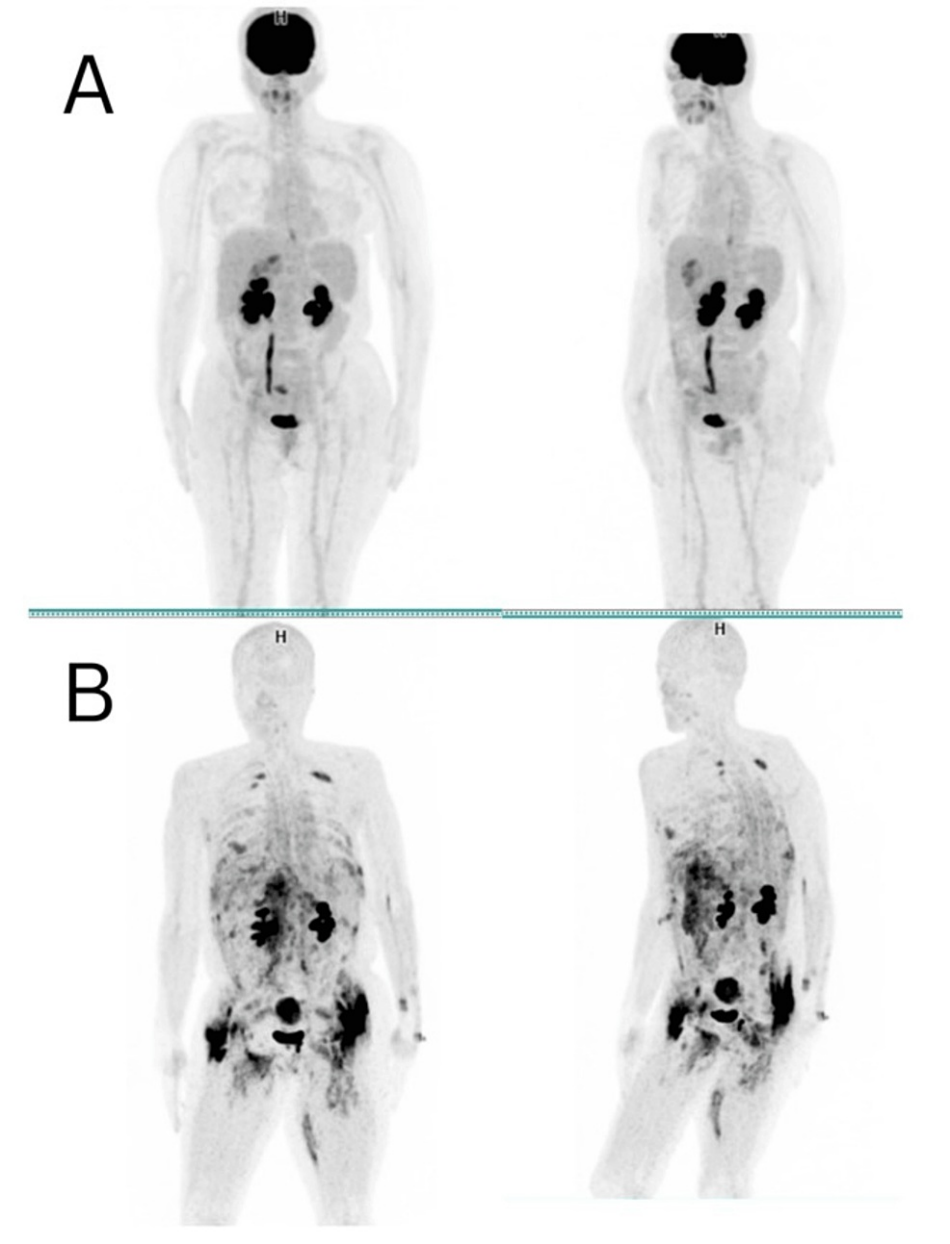
MIP images: (A) baseline [18F]-FDG PET/CT scan and (B) follow-up [68Ga]-FAPI PET/CT image MIP: maximum intensity projection

The initial workup included comprehensive biochemical tests encompassing a complete blood count, renal function test, liver function test, serum electrolytes, carcinoembryonic antigen (CEA), cancer antigen (CA) 19-9, and CA 125, revealing a slight increase in the level of CEA and normocytic anemia (Table 1). [18F]-FDG PET scan depicted gastric antrum and pylorus thickening with uterine metabolic activity, non-[18F]-FDG avid upper abdominal lymph nodes, lung nodules, and pelvic cystic lesions (Figure 1A, 1B).

**Table 1:** Biochemical profile of patient at initial presentation Hb: hemoglobin; PCV: packed cell volume; RBC: red blood cell; MCV: mean corpuscular volume; MCH: mean corpuscular hemoglobin; MCHC: mean corpuscular hemoglobin concentration; WBC: white blood cell; MPV: mean platelet volume; RDW: red cell distribution width; ALT: alanine aminotransferase; AST: aspartate aminotransferase; CEA: carcinoembryonic antigen

Test	Result/status	Units	Reference range
Hb	10.6	g/dL	12-16
PCV	30.3	%	36-45
RBC	3.59	10^6^/uL	3.9-5.2
MCV	84.4	fl	80-94
MCH	30.1	pg	27-31
MCHC	35.6	g/dL	32-36
WBC	5.25	10^3^/uL	4-11
Platelet count, blood	340	10^3^/uL	150-400
MPV	10.0	fl	7.5-9.3
RDW	20.0	%	11.6-14.6
Cancer antigen 125	24.40	U/mL	0-35
Hepatitis B core Ab total	2.340 (negative)	IU/mL	Ref: positive: <0.8 COI, grey zone: ≥0.8 to <1.2 COI, negative: >1.2 COI
Hepatitis C virus Ab	0.039 (negative)	IU/mL	Ref: negative: <0.8 COI, grey zone: ≥0.8 to <1.2 COI, positive: >1.2 COI
Hepatitis B surface Ab	<2.0 (negative)	IU/mL	Ref: positive: ≥10 IU/L, negative: <10 IU/L
Hepatitis B surface An	0.456 (negative)		Ref: negative: <0.8 COI, grey zone: ≥0.8 to <1.2 COI, positive: >1.2 COI
Creatinine	0.9	mg/dL	0.5-1.12
Urea	14.60	mg/dL	12-36
Uric acid	4.19	mg/dL	2.2-6.4
Sodium	139.10	mmol/L	135-145
Potassium	3.7	mmol/L	3.6-5.2
Chloride	105.70	mmol/L	97-110
Total protein	7.6	g/dL	6.2-8.2
Albumin	4	g/dL	4-5.1
ALT	22.8	U/L	7-47
AST	29.60	U/L	8-48
Alkaline phosphatase	64.400	U/L	37-130
Bilirubin total	0.61	mg/dL	0.28-1
Bilirubin direct	0.27	mg/dL	0.01-0.3
Calcium	9.49	mg/dL	8.8-10.2
Adjusted calcium	9.73	mg/dL	8.8-10.2
Magnesium	1.88	mg/dL	1.7-2.4
Phosphorus	2.59	mg/dL	2.5-4.5
Bicarbonate	21.1	mmol/L	21.1
CEA	4.8	ng/mL	0-2.9

**Figure 2:**
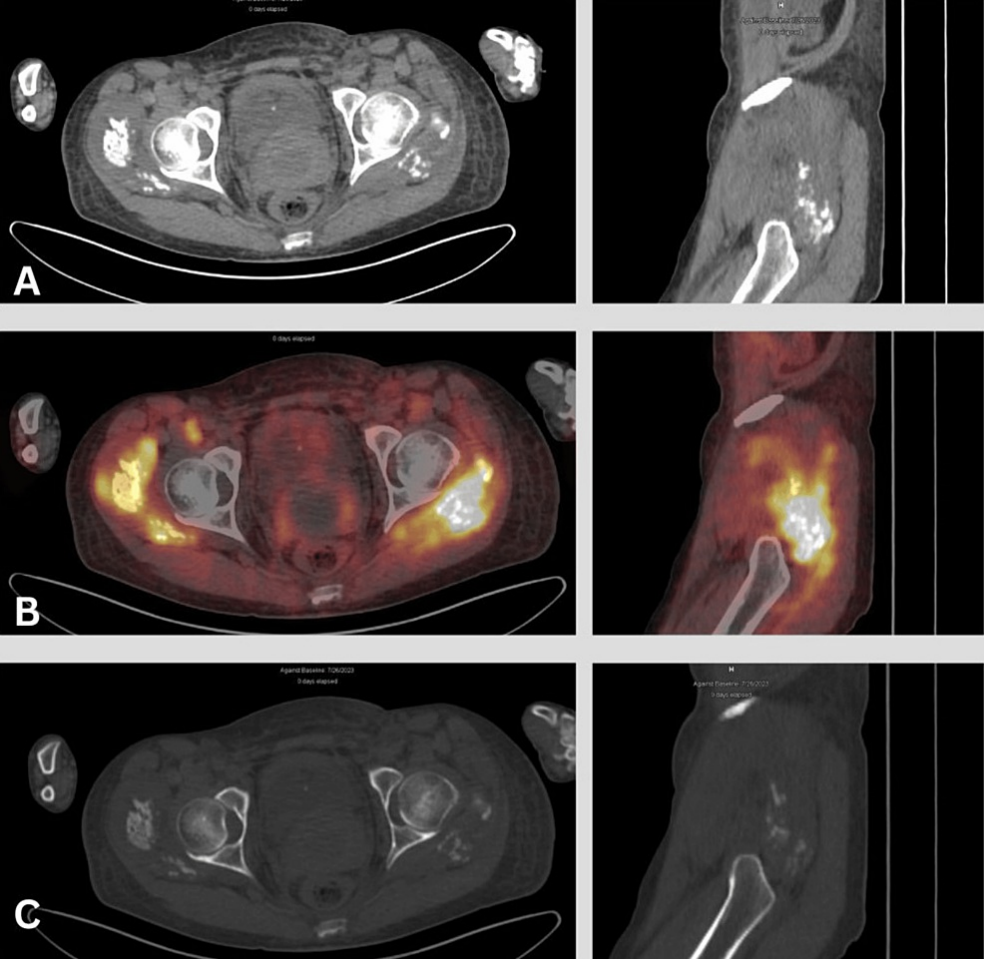
[68Ga]-FAPI PET/CT images Follow-up [68Ga]-FAPI PET/CT images. (A) CT soft tissue window axial and left sagittal views. (B) Fused PET/CT axial and left sagittal views. (C) CT bone window axial and left sagittal views reveal extensive soft tissue ossification surrounding the bilateral hip joints, mainly involving the gluteus minimus and medius muscles. These ossifications exhibit a distinctive pattern, characterized by peripheral mature lamellar bone and a central region consisting of immature bone, demonstrating intense [68Ga]-FAPI activity with a maximum standardized uptake value (SUVmax=18.3) within the left gluteal area

The patient underwent neoadjuvant fluorouracil, oxaliplatin, and docetaxel (FLOT) chemotherapy, followed by subtotal gastrectomy with extended systemic lymphadenectomy (D2 lymphadenectomy). Residual disease was confirmed on CT, prompting adjuvant FLOT chemotherapy. Subsequent management involved a multidisciplinary team (MDT)-guided follow-up utilizing a [68Ga]-FAPI PET scan. The scan revealed minimal [68Ga]-FAPI activity in the surgical site, likely indicative of postoperative reactive changes secondary to inflammation. Furthermore, [68Ga]-FAPI avid metastases were detected, including bilateral pleural effusion, moderate abdominopelvic ascites, and peritoneal/omental thickenings (Figure 2).

**Figure 3:**
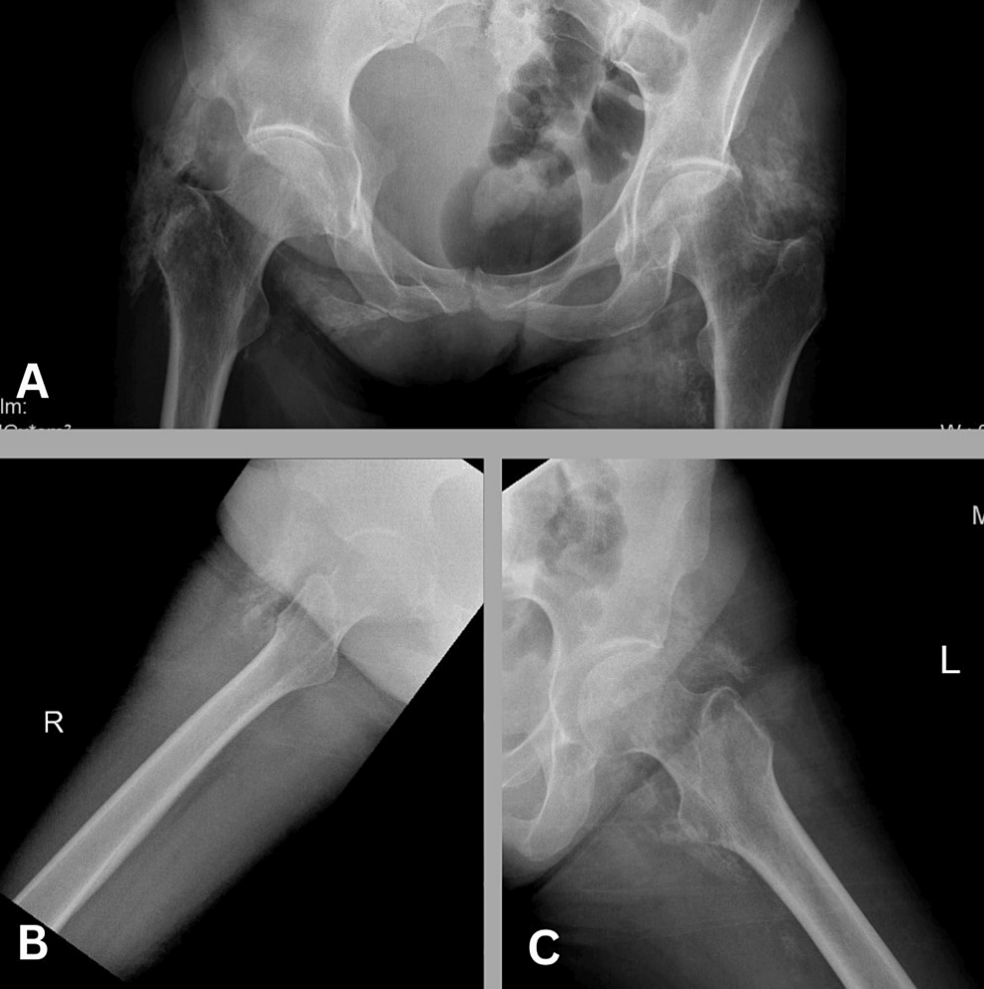
Radiographs of both hip joints Anteroposterior (A), right lateral (B), and left lateral (C) radiographs of both hip joints illustrate the presence of multiple mature heterotopic ossifications encircling both hip joints. These ossifications extend superiorly, in close proximity to the inferior aspect of the ilium, and inferiorly, just below the level of the lesser trochanter

Significantly, there was an unexpected occurrence of significant intramuscular ossifications/calcifications in the pelvic and bilateral thigh muscles, demonstrating a high affinity for [68Ga]-FAPI (Figure 3). Multiple mature heterotopic ossifications were observed encircling both hip joints on the radiographs.

A comprehensive assessment was carried out, which included a detailed review of the patient's medical records, a thorough physical examination, and a comprehensive analysis of laboratory tests. While the patient denied any previous trauma, localized tenderness in relevant anatomical regions and restricted hip extension were observed upon examination. Furthermore, the diagnostic approach integrated functional and anatomical data obtained from [68Ga]-FAPI PET/CT and radiographic evaluations. Based on multimodal imaging findings, the patient received a confirmed diagnosis of MO, likely idiopathic in origin.

## Discussion

MO is a rare condition characterized by the abnormal formation of bone tissue in soft tissues located outside the skeletal system. The clinical presentation of MO is often nonspecific and can appear as a rapidly growing, tender mass that closely resembles more serious medical diseases [13]. The anatomic origin of MO displays variability, with a predominance of approximately 80% of cases localizing within the substantial musculature of the extremities. In general, the clinical signs of MO strongly resemble those of aggressive pathological neoplasms, like sarcomas, necessitating a high level of clinical suspicion for accurate diagnosis [13].

The diagnosis of MO heavily relies on thorough history-taking, particularly in identifying any antecedent traumatic events, as well as a comprehensive assessment of clinical symptoms. Confirmation of diagnosis is made easier by using radiological imaging and histological analysis, particularly when the existence of typical zone phenomena can be shown [13].

The utility of FAPI imaging is increasingly recognized in evaluating inflammatory and infectious diseases [14]. Reports suggest that patients with immune-mediated inflammatory diseases, characterized by persistent inflammation and tissue responses, show localized radiolabeled [68Ga]-FAPI uptake, indicating active tissue remodeling [14,15]. Consequently, it is not uncommon for benign lesions to exhibit [68Ga]-FAPI uptake, with bones and joints frequently being the locations where such findings are observed [14].

Multiple case studies have provided evidence that the uptake of radiolabeled FAPI is associated with a significant number of non-cancerous disorders that especially affect the bones and joints, including ankylosing spondylitis and fibrous dysplasia [13].

Molecular imaging has been pivotal in the evaluation of multiple cases of MO following traumatic incidents [16]. Sasaki et al. documented a notable case involving multiple traumatic MOs localized within the masseter, triceps brachii, and biceps brachii muscles. This was effectively identified using [18F]-FDG-PET/CT scans, which demonstrated calcification sites characterized by minimal [18F]-FDG uptake. The SUVmax values recorded were comparable to those of liver references, albeit slightly elevated above the baseline muscular uptake [16]. In our reported case, no increased [18F]-FDG uptake was noted at the trauma sites. Another occurrence of MO at a hematoma site was evaluated using two distinct radiotracers: [18F]-FDG PET/CT and [99mTc]-hydroxymethylene-diphosphonate skeletal scintigraphy [17]. Both imaging modalities revealed only mild tracer uptake [18]. More recently, the advent of [68Ga]-FAPI imaging has introduced new dimensions to the diagnostic process in nuclear oncology [18]. A particularly intriguing case involved a 72-year-old male patient who experienced persistent right hip pain, ambulatory difficulties, and a palpable mass in the right hip over a three-month period [19]. Initial CT imaging suggested osteonecrosis of the right femoral head alongside a calcified mass at the hip. Given the clinical presentation and radiologic findings, a neoplastic process was considered. Consequently, the patient was enrolled in a clinical trial evaluating [68Ga]-FAPI. The [68Ga]-FAPI PET/CT scan revealed significant [68Ga]-FAPI activity within the right hip mass. Subsequent surgical intervention and pathological examination of the right hip confirmed the presence of MO [19].

Up to our knowledge, this is the first case report specifically addressing bilateral [68Ga]-FAPI uptake in bilateral gluteal MO and one of the rarest instances where [68Ga]-FAPI uptake has been documented in the context of MO.

In our specific case, MO has demonstrated the potential to exhibit robust [68Ga]-FAPI activity, which closely resembles the pattern seen in osteosarcoma [20]. Consequently, it is imperative to include MO as a viable consideration when confronted with the diagnostic challenge of soft tissue calcifications featuring intense [68Ga]-FAPI uptake.

## Conclusions

In this unique case, we identified elevated [68Ga]-FAPI uptake in both gluteal muscles, indicative of MO upon radiological examination. While this uptake bears resemblance to patterns observed in osteosarcoma, it's essential to recognize that similar uptake can manifest in benign bone and joint conditions. Therefore, there's no cause for concern regarding malignancy in this context.
